# A Simple Technique to Manage Anxiety During Tooth Extraction

**DOI:** 10.7759/cureus.29275

**Published:** 2022-09-17

**Authors:** Ramesh Kunusoth, Shreya Colvenkar, Aditya Mohan Alwala, Sneha Bharadwaj, Ramanarayana Boyapati

**Affiliations:** 1 Department of Oral and Maxillofacial Surgery, MNR Dental College and Hospital, Sangareddy, IND; 2 Department of Prosthodontics, MNR Dental College and Hospital, Sangareddy, IND; 3 Department of Prosthodontics, Malla Reddy Dental College for Women, Hyderabad, IND; 4 Department of Oral and Maxillofacial Surgery, Sibar Institute of Dental Sciences, Guntur, IND

**Keywords:** local anesthesia, extraction, syringe, virtual reality heaset, anxiety

## Abstract

Pain‐free dental treatment is important for reducing anxiety, treatment completion, and enhancing acceptance of future dental treatment. Injectable local anesthesia assists this pain‐free approach but can cause anxiety in few patients. Achieving proper anesthesia is important to complete the procedure successfully, thereby adding to patient's care and comfort. This article describes a simple technique to manage dental anxiety using virtual reality headset. Audio visual aid helped the patient to relax and eliminate the phobia of local anesthesia injection during surgical tooth extraction. The simple technique enhanced the patient’s ability to continue the dental procedure.

## Introduction

Dental anxiety is an unpleasant emotional reaction to certain procedures in a dental hospital. High levels of anxiety and/or fear are felt among one out of every seven patients treated in western countries [1]. Anxious patient either skip the appointment or delay the treatment [2]. When a patient avoids regular dental visits out of fear, their oral health condition worsens. This translates to patient visiting dental office once the pathology is at advanced stage, requiring more complex treatment. More complex treatment reinforces or exacerbates the fear, which leads to continued avoidance [3].

Local anesthetic injection is associated with high levels of anxiety in few patients [4,5]. This extreme anxiety can make simple dental procedures like extraction time-consuming. Achieving proper anesthesia is essential to complete the procedure successfully, thereby adding to the patient's care and comfort.

Various techniques have been mentioned in the literature to manage dental anxiety which can be broadly divided into pharmacological [3] and non-pharmacological management [6-11]. Non-pharmacological management includes communication skills, rapport and trust building, systemic desensitization, acupressure, laser acupuncture, hypnosis, music, audio-visual aid, guided imagery, meditation, relaxation, breathing, and virtual reality headset. This article describes a simple straightforward technique to manage needle phobia during extraction with virtual reality headset. The virtual reality headset diminished the fear and anxiety, allowing the extraction to be carried out smoothly.

## Case presentation

**Figure 1:**
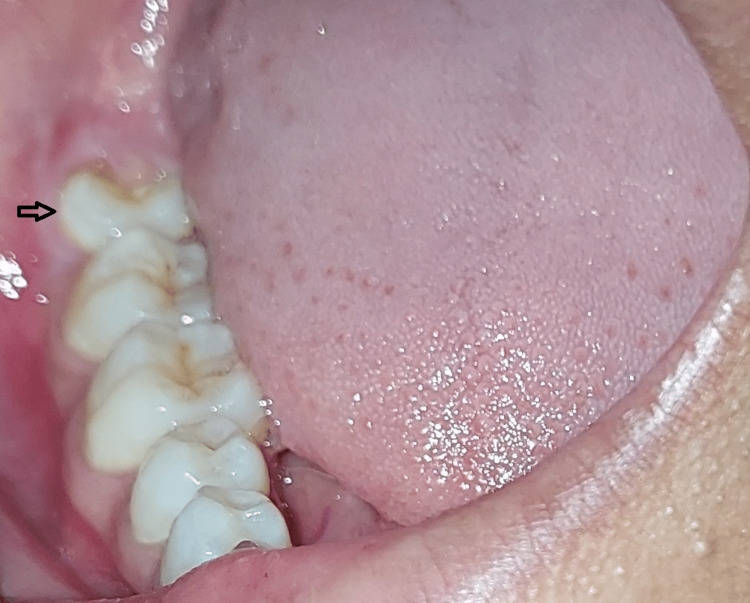
Partially erupted third molar of the patient.

A 20-year-old female patient reported to the Department of Oral and Maxillofacial Surgery for extraction of mesioangular impacted mandibular right third molar. The patient complained of pain and food impaction in relation to the impacted tooth. Intraoral examination revealed partially erupted tooth in the oral cavity (Figure 1).

The word extraction made the patient anxious, and she totally denied the procedure. History revealed that she has a phobia of local anesthesia injection and had a traumatic experience of extraction during childhood. The patient was counseled that the procedure will be pain-free. The patient's blood pressure and heart rate were monitored before extraction.

The patient was asked to do meditation and deep breathing. Once patient was relaxed, distraction technique using music and audio-visual media was used to inject local anesthesia. But the distraction technique failed. Again, the patient was relaxed by peaceful conversation and meditation.

**Figure 2:**
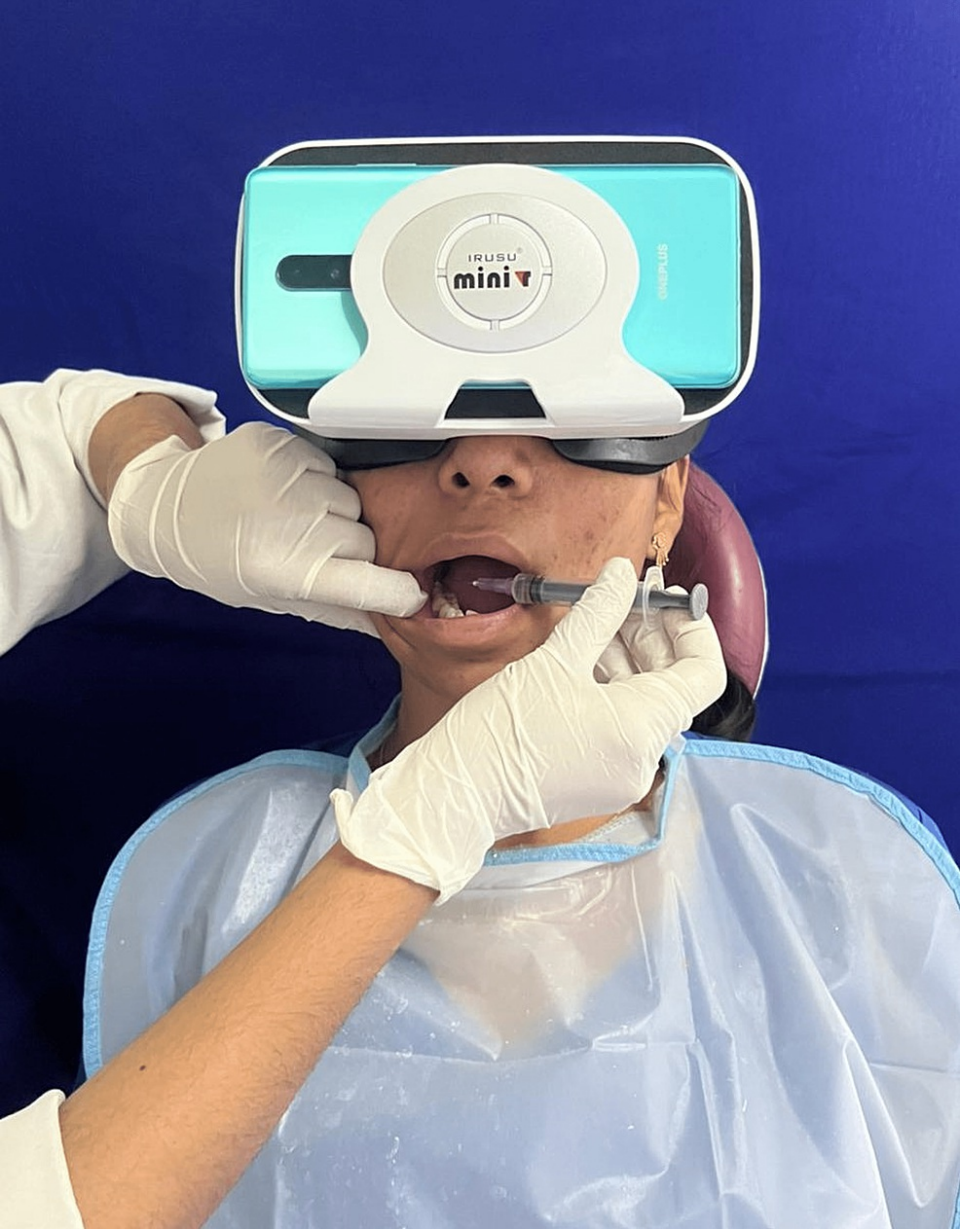
Patient with virtual reality headset.

Since the mere site of syringe created anxiety in the patient, it was decided to alter the patient’s vision by using virtual reality headset. The patient was instructed to wear virtual reality headset and soothing video of the patient’s choice was played (Figure 2).

**Figure 3:**
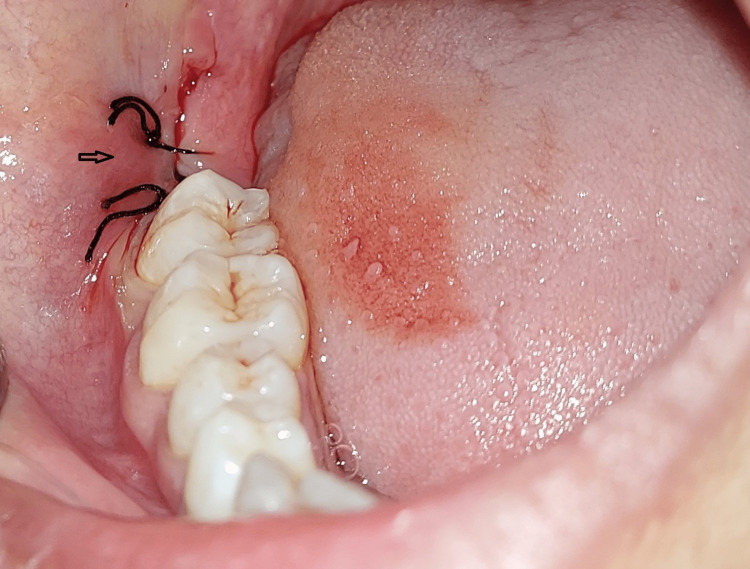
Impacted third molar tooth extracted and sutured.

The headset temporarily blocked the patient from outside world. The local anesthetic gel was applied followed by light massage in that area. Under local anesthesia, tooth extraction was carried out and extracted site was sutured (Figure 3). Virtual reality headset was removed on completion of procedure.

The patient's blood pressure and heart rate were immediately recorded after tooth extraction. The patient was given post-extraction instructions and medications. The patient was surprised that procedure was completed very fast and without pain. During recall, the extraction site showed signs of good healing.

## Discussion

Dental anxiety makes simple surgical procedures time-consuming and difficult. It is very important to take complete history to understand the underlying cause of dental anxiety. The patient presented with traumatic extraction during childhood. The unpleasant previous dental experiences created a lot of anxiety during subsequent dental visits. To successfully manage dental anxiety, the patient was received with a calm and patience attitude.

There are various techniques mentioned in the literature for the management of anxiety [6-9]. Among all, distraction technique is more commonly and successfully used for short dental procedures [6-8]. The distraction techniques using music and audio-visual media did not help in this situation [7,8]. Hence virtual reality headset was tried. Virtual reality headset is a powerful distraction tool for pain as well as anxiety [9-11]. Virtual reality provides a 3D environment (including both computer graphics and 360-degree video) that surrounds a user and responds to an individual’s actions in a natural way. Virtual reality headsets help a user enjoy an immersive 3D environment by eliminating their connection with the real world. Virtual reality headsets can be used with myriad of apps on smartphones to provide a virtual reality experience like sitting on a beach. It works by engaging different senses like hearing and vision together and diverting attention from painful stimuli to a new tranquil 3D environment. It takes the patient into virtual environment and creates a sense of real presence in that environment.

Qin et al. demonstrated that virtual reality is an effective distraction method to relieve anxiety and pain during tooth extraction. They hypothesized that virtual reality headset can control the elevation of blood pressure and heart rate in patients with hypertension. In the present case, the patient’s blood pressure and pulse rate were high before extraction but soon after extraction, it was back to normal which was in line with the study conducted by Qin et al. [10].

Ougradar and Ahmed concluded that virtual reality technology plays a beneficial role in reducing anxiety levels in most patients (65%) while undergoing dental extractions. Virtual reality distraction during dental extraction created positive experience, leading to greater willingness to return for treatment in the future. Using virtual reality headset helped the patient to break the cycle of negative experiences. The patient complained of no pain during extraction and agreed to regular dental visits [11].

No difficulty was faced during mandibular extraction, but a larger size headset can be a challenge during maxillary extraction. This can be managed by using a smaller headset. The cost of a headset varies from 15 to 130 dollars which is a good one-time investment for dental setup. The dentist can peacefully carry out short dental procedures like extraction very easily.

## Conclusions

The distraction technique using virtual reality headset was very effective in controlling the dental phobia during extraction. The patient was very happy that the procedure was smoothly completed without pain. Virtual reality headset increased the patient’s confidence for regular dental checkups.
